# Mathematical Abilities in School-Aged Children: A Structural Magnetic Resonance Imaging Analysis With Radiomics

**DOI:** 10.3389/fnins.2022.819069

**Published:** 2022-04-14

**Authors:** Violeta Pina, Víctor M. Campello, Karim Lekadir, Santi Seguí, Jose M. García-Santos, Luis J. Fuentes

**Affiliations:** ^1^Departamento de Psicología Evolutiva y de la Educación, Facultad de Educación, Economía y Tecnología de Ceuta, Universidad de Granada, Ceuta, Spain; ^2^Departament de Matemàtiques i Informàtica, Universitat de Barcelona, Barcelona, Spain; ^3^Servicio de Radiología, Hospital Morales Meseguer, Murcia, Spain; ^4^Departamento de Psicología Básica y Metodología, Universidad de Murcia, Murcia, Spain

**Keywords:** school-aged children, machine learning, mathematical performance, sMRI, radiomics

## Abstract

Structural magnetic resonance imaging (sMRI) studies have shown that children that differ in some mathematical abilities show differences in gray matter volume mainly in parietal and frontal regions that are involved in number processing, attentional control, and memory. In the present study, a structural neuroimaging analysis based on radiomics and machine learning models is presented with the aim of identifying the brain areas that better predict children’s performance in a variety of mathematical tests. A sample of 77 school-aged children from third to sixth grade were administered four mathematical tests: Math fluency, Calculation, Applied problems and Quantitative concepts as well as a structural brain imaging scan. By extracting radiomics related to the shape, intensity, and texture of specific brain areas, we observed that areas from the frontal, parietal, temporal, and occipital lobes, basal ganglia, and limbic system, were differentially related to children’s performance in the mathematical tests. sMRI-based analyses in the context of mathematical performance have been mainly focused on volumetric measures. However, the results for radiomics-based analysis showed that for these areas, texture features were the most important for the regression models, while volume accounted for less than 15% of the shape importance. These findings highlight the potential of radiomics for more in-depth analysis of medical images for the identification of brain areas related to mathematical abilities.

## Introduction

Recent research has focused on determining the cognitive processes that are associated with mathematical performance, as well as the brain areas involved. The goal is not only to achieve a more comprehensive understanding of how children and adults solve mathematical problems, but also to better characterize the disorders that affect mathematical abilities.

There is now ample evidence demonstrating the existence of crucial brain areas related to mathematical abilities. For instance, voxel-based morphometry (VBM) studies have found that children that differ in some mathematical abilities show differences in gray matter volume in several brain areas related to number processing, attentional control, and memory, such as the posterior parietal cortex, left intraparietal sulcus, right fusiform gyrus, areas of the frontal cortex such as the inferior frontal gyrus and the middle frontal gyrus, the hippocampus, and occipito-temporal cortex (Li et al., 2013; Evans et al., 2015; Price et al., 2016; Peters and De Smedt, 2018; Fritz et al., 2019; Polspoel et al., 2020, 2021). The relationship between domain-general cognitive abilities (e.g., attention, working memory) and mathematical performance has been widely established in behavioral meta-analyses (Friso-van den Bos et al., 2013; Peng et al., 2016), computerized working memory-based interventions at school (Sánchez-Pérez et al., 2018a), and fMRI studies (Rotzer et al., 2009; Dumontheil and Klingberg, 2011; Ashkenazi et al., 2013; Metcalfe et al., 2013).

In the present study, we adopted a radiomics-based approach to further explore which structural image features are better predictors of children’s performance in a variety of mathematical abilities. Radiomics refers to a type of image analysis mainly applied in the field of precision medicine, that allows researchers to perform a more exhaustive analysis of medical images, by computing and mining a large pool (thousands) of advanced imaging features (Gillies et al., 2016). Radiomic features include shape and first and second order texture features. Shape-based features describe geometric properties of regions of interest (ROIs). These features include compactness and sphericity, which describe how the shape of a ROI differs from that of a circle or a sphere. First-order texture features refer to commonly used histogram statistics that describe ROI intensity distributions, such as mean, median, kurtosis, skewness or entropy, among others. Second-order texture features describe statistical interrelationships between voxels with similar intensity values. In particular, these features account for spatial characteristics of an image in terms of intensity values such as coarseness, heterogeneity, symmetry and variability (Larroza et al., 2016; Park and Kim, 2018; Mayerhoefer et al., 2020).

So far, radiomics-based analyses have been carried out mostly in oncology and more recently in lung and cardiovascular applications (Huang et al., 2016; Guerrisi et al., 2020; Raisi-Estabragh et al., 2020). To date, few studies have explored this technique in the field of cognitive disorders (Sun et al., 2018; Cui et al., 2021), and importantly, texture features allowed researchers to better differentiate between clinical and typical populations and revealed as the most appropriate biomarkers for diagnosis. sMRI-based analyses in the context of mathematical performance have been focused primarily on volumetric measures. By assessing volume only (as in VBM), a great proportion of information available in the image is usually ignored, including everything that relates to intensity values. As an example, in neurodevelopmental pathologies with severe cognitive symptoms, volume may remain within the normal range, or the differences observed may also be associated with other pathologies (Koolschijn et al., 2009). We hypothesized that additional information extracted from structural images of typically developing children regarding shape, intensity, and especially texture, can aid in the identification of areas related to mathematical performance. Therefore, the objective of the present study is twofold. First, we sought to replicate and extend the brain areas associated to different mathematical abilities exhibited by typically developing children, in accordance with the results of previous studies stemming from the use of neuroimaging techniques. Second, to perform a more in-depth analysis of the images extracted from the sMRI protocol, based on radiomics, to determine which features of the brain areas are the best predictors of mathematical abilities in school-aged children.

## Materials and Methods

### Participants

One hundred and four 7–12-year-old typically developing children that participated in a larger project (Sánchez-Pérez et al., 2018a), took part in the present neuroimaging study. Children were recruited from two primary education schools in the Región de Murcia (Spain), and were enrolled in grades 3–6. Data were collected from mathematical standardized tests and sMRI. The final sample was reduced to 77 children (43 boys and 34 girls, mean age 9.7; SD 1.2) after excluding: data with excessive motion (by looking qualitatively at each volume), children that refused to enter into the scanner at the moment of scanning, and equipment failures. The project was approved by the Ethics Committee of the University of Murcia and it was conducted in accordance with the approved guidelines and the Declaration of Helsinki. Written informed consent was obtained from the parents, and oral consent was obtained from the children at the moment of scanning.

### Behavioral Data

Children’s math abilities were assessed through the Woodcock-Johnson III (WJ-III) Achievement battery for children aged 6–13 years in Spain (Diamantopoulou et al., 2012). The battery is composed of four tests: Math fluency, Calculation, Applied problems, and Quantitative concepts. Descriptive data are shown in Table 1.

**TABLE 1:** Descriptors for the children’s characteristics and the mathematical ability tests considered in this study.

			Math fluency		Calculation		Applied problems		Quantitative concepts	
Grade	Boys/girls	Age	Score	Est. grade	Score	Est. grade	Score	Est. grade	Score	Est. grade
3	9/15	8.5 (0.33)	37.6 (6.9)	2.8	13.0 (2.4)	3.3	29.7 (3.0)	3.0	16.9 (2.1)	3.4
4	15/9	9.4 (0.39)	46.5 (11.3)	3.6	16.8 (2.1)	4.7	33.8 (4.4)	4.1	19.4 (2.8)	4.7
5	12/5	10.6 (0.37)	62.4 (18.6)	5.0	18.4 (1.8)	5.4	35.8 (3.5)	4.7	20.6 (1.6)	5.1
6	7/5	11.6 (0.27)	63.3 (19.6)	5.1	19.3 (2.1)	5.9	39.3 (5.2)	6.1	22.8 (2.1)	6.4
All	43/34	9.7 (1.15)	49.9 (17.2)	–	16.3 (3.2)	–	33.8 (5.1)	–	19.4 (3.0)	–

*Values are presented as mean (standard deviation).*

*Est. grade, estimated grade mean provided by each test.*

The Math fluency test measures the ability to quickly solve a total of 160 simple addition, subtraction, and multiplication within 3 min. The Calculation test measures the ability to perform mathematical computations and consists of 46 items of ascending difficulty involving addition, subtraction, multiplication, division, rational number arithmetic, trigonometry, algebra, and calculus. The Applied problems test measures the ability to analyze and solve 62 ascending difficulty math word problems. The Quantitative concepts test measures the knowledge about mathematical concepts, symbols, vocabulary and numerical series, and consists of 57 items (Pina et al., 2015; Sánchez-Pérez et al., 2018a). Each item represented one point if the child answered correctly. The maximum score for each test is the number of items. Partial correlations analyses, controlled by age, between Math fluency, Calculation, Applied problems and Quantitative concepts scores were all positive and ranged from 0.34 to 0.69 (see Table 2).

**TABLE 2:** Partial correlations between the Woodcock Johnson III mathematical tests controlled by age.

	Calculation	Applied problems	Quantitative concepts
Math fluency	0.386*	0.344*	0.360*
Calculation		0.503**	0.498**
Applied problems			0.686**

*p < 0.01, **p < 0.001.

### Image Acquisition

Anatomical MRI data were acquired using a General Electric 1.5 T HDX scanner located at the Hospital General Universitario Morales Meseguer (Murcia, Spain). A parent was present with the child during the scanning session and earplugs were used for protecting the child’s hearing. Soft pads were also used to reduce motion artifacts. The sequence parameters was: TR, 12.4 ms; TE, 5.2–15 ms; voxel size, 1 × 1 × 1 mm; flip angle, 12°; 142 axial slices (Sánchez-Pérez et al., 2019).

### Image Analysis

**FIGURE 1:**
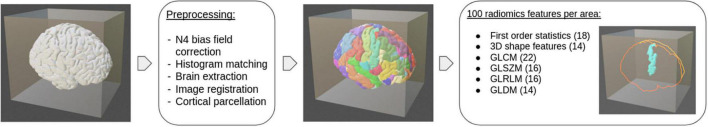
Schematic pipeline for extracting radiomics features from sMRI.

Several pre-processing steps were conducted prior to the cortical parcellation and features extraction (see Figure 1).

First of all, possible low frequency intensity inhomogeneities were corrected using N4 Bias Field (SimpleITK, version 1.2.4, Lowekamp et al., 2013). Then, image intensities were standardized using histogram matching for the whole volume with a reference participant selected visually (scikit-image, version 0.18.1). Finally, three-dimensional image registration was used to transform the images to a common space with Advanced Normalization Tools (ANTs, version 0.2.2, Tustison et al., 2014) for Python. The pre-processed images were then used to extract and parcellate the brain with the Freesurfer package (version 6, Fischl et al., 2004) according to the Destrieux Atlas (Destrieux et al., 2010). The parcellated ROIs were transformed back into native space for radiomics feature extraction. Within each of the 191 brain areas, a set of 100 radiomics features were computed accounting for its shape, intensity and texture using the PyRadiomics library (version 2.2.0, van Griethuysen et al., 2017) with the default configuration (bin width of 25). See Supplementary Table 1 for the list of extracted features.

### Machine Learning-Based Area Ordering

To determine the main brain areas involved in each mathematical test a regression-based analysis, with age as a control variable, was proposed to predict the final test scores. For each regression analysis and for each brain area, a prediction error was extracted using the mean absolute deviation of the actual score (Mean Absolute Error, MAE). This allowed us to rank the brain areas from the most predictive to the least predictive. MAEs were also computed for regression analyses using only age as input variable to assess the added value of radiomics features. MAE was considered a more appropriate measure than the mean squared error (MSE) due to its robustness to deal with outliers.

A Random Forest (RF) regression model is proposed to account for the linear and non-linear relationships of brain areas characteristics with mathematical abilities. A RF is a combination of decision trees built from dataset sub-samples that uses averaging of the individual predictions for improving overall accuracy and reducing overfitting to the training sample (Friedman et al., 2001). For this reason and motivated by its simplicity and its wide usage, RF was selected over other options in the present study. A set of hyperparameters need to be selected prior to model training. For the current research, all parameters were chosen as default but for the maximum number of decisions that each tree can make. This value was set to 5 to prevent decision trees from being too specific to the training set and to reduce overfitting. By default, the number of trees was 100 (scikit-learn, version 0.22.1).

**FIGURE 2:**
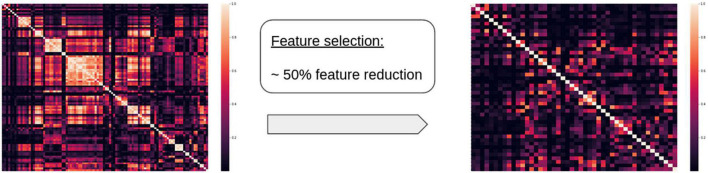
Example of a correlation matrix for radiomics features before and after the feature reduction for a given area, showing significant reduction in the number of correlated features.

Due to our rather small sample size and the large input feature space, a feature selection was conducted for each brain area during training. Variables with a squared correlation coefficient higher than 0.8 (Pearson —ρ— ≈ 0.9) between them were assumed to share similar information and only one was preserved. Comparisons were performed sequentially following the order established in Supplementary Table 1, i.e., at step *i* the feature with index *i* was compared against all others and those with high correlation were removed, while preserving most of the shape features. This ensured that any texture feature that showed a high correlation with shape features was removed (see Figure 2, for illustration). This method of feature selection was selected over other alternatives because of its simplicity and the interest in linearly eliminating redundant features. The final selected features accounted for a percentage between 81 and 39% of the initial features, depending on the area under consideration (see Supplementary Figure 1 for further details). The selected features, together with age, were entered as input variables in the correlation analyses.

**FIGURE 3:**
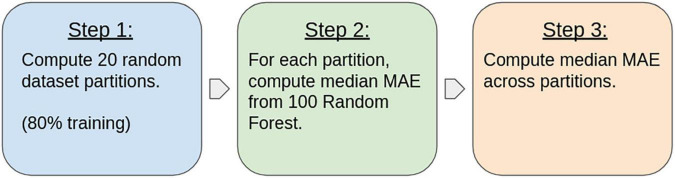
Method pipeline used for computing the resulting errors for each area and for each mathematical test.

For each test and each ROI, the following pipeline was used to ensure the robustness of the final results (see Figure 3): (1) 20 random partitions of the dataset in training (80%) and testing (20%) were generated, (2) 100 different RF regression models were trained on each partition to obtain an average MAE for the hold-out validation set, and (3) the resulting MAE was obtained as the median across the 20 partitions. The median was chosen over the mean due to its robustness against outliers.

Finally, the contribution of each family of features was assessed for the selected models by computing the Gini *importance* (also called mean decrease of impurity importance). The Gini *importance* is a measure derived from the training of each model and indicates how often a particular feature was selected for a split, and how large its overall discriminative value was for the problem under study (Menze et al., 2009).

## Results

To explore any effects of our independent variables (age and sex) on children’s mathematics performance, we conducted two-way ANOVAs with age and sex as between-participants factors and scores on each mathematics test as the dependent variables. The results showed that age produced statistically significant main effects in all mathematics tests, Math fluency [*F*(4,67) = 8.3, *p* < 0.001, η*p*^2^ = 0.33], Calculation [*F*(4,67) = 9.77, *p* < 0.001, η*p*^2^ = 0.37], Applied problems [*F*(4,67) = 6.48, *p* < 0.001, η*p*^2^ = 0.28], and Quantitative concepts [*F*(4,67) = 7.57, *p* < 0.001, η*p*^2^ = 0.31].

[*F*(4, 67) = 8.3, *p* < 0.001], Calculation [*F*(4, 67) = 9.77, *p* < 0.001], Applied problems [*F*(4, 67) = 6.48, *p* < 0.001], and Quantitative concepts [*F*(4, 67) = 7.57, *p* < 0.001]. In contrast, sex did not produce any significant main effect, nor did it interact with age in any of the mathematics tests (all *ps* > 0.05). These results would be in line with the results of several meta-analyses that have found no sex differences in mathematics performance at the behavioral level (e.g., Lindberg et al., 2010).

For each mathematical test the most predictive and significant areas were selected. To do this, the resulting MAEs were assumed to follow a normal distribution and those areas with an error below two standard deviations from the mean (*p* < 0.022) were classified as “the most relevant.” To assess whether the RF regression model prediction was significantly better than random, the *p*-value associated with the selected areas was obtained using a non-parametric randomization test. In this test random features were provided as input for the regression models to extract the noise distribution (Manly and Navarro Alberto, 2020). A similar approach was proposed by He et al. (2020) to find significant brain biomarkers related to inhibitory control, using sMRI data. Results below two standard deviations from the noise distribution mean (*p* < 0.022) were classified as significant. Multiple test comparison corrections were not applied in this study because the initial hypothesis refers to the relationship of individual areas with mathematical tests and as such, falls into the domain of individual testing (Rubin, 2021). Table 3 summarizes the final selected areas for each test. The added value of the feature selection step is demonstrated in Supplementary Table 2 by comparing the drop in accuracy of the predictive areas.

**TABLE 3:** Predictive areas of math performance in the four tests of the WJ-III battery.

Test	Atlas label	H	Loc	Area name—Acronym	*P*-value (Rand. test)	MAE
Math fluency	12,140	R	IF	Vertical ramus of the anterior segment of the lateral sulcus (or fissure)—ASLS	1.2 × 10-7	0.210
	12,154	R	MF	Middle frontal sulcus—MFS	4.7 × 10-7	0.212
	11,157	L	PL	Intraparietal sulcus and transverse parietal sulci—IPS	6.1 × 10-7	0.212
	12,128	R	PL	Post-central gyrus—PSTCG	8.1 × 10-6	0.216
	11,154	L	MF	Middle frontal sulcus—MFS	1.3 × 10-5	0.217
Calculation	26	L	BG	Nucleus accumbens—NA	1.5 × 10-3	0.116
	11,108	L	LS	Middle-posterior part of the cingulate gyrus and sulcus—PCG	3.6 × 10-3	0.118
	12,171	R	FL	Suborbital sulcus (sulcus rostrales, supraorbital sulcus)—SS	4.0 × 10-3	0.118
	12,111	R	OL	Cuneus gyrus—CG	4.0 × 10-3	0.118
	12,128	R	PL	Post-central gyrus—PSTCG	5.1 × 10-3	0.118
Applied problems	12,131	R	IF	Straight gyrus, Gyrus rectus—SG	7.4 × 10-10	0.106
	12,113	R	IF	Orbital part of the inferior frontal gyrus—OIFG	1.2 × 10-5	0.112
Quantitative concepts	11,123	L	LS	Parahippocampal gyrus, parahippocampal part of the middle occipito-temporal gyrus—PHPG	3.0 × 10-4	0.098
	11,125	L	PL	Angular gyrus—AG	1.7 × 10-3	0.100
	11,133	L	TL	Anterior transverse temporal gyrus (of Heschl)—HG	2.8 × 10-3	0.100

*H and Loc stand for Hemisphere and Localization, respectively. MAE, Mean absolute error. Localization: IF, inferior Frontal; MF, middle frontal; PL, parietal lobe; BG, basal ganglia; LS, limbic system; FL, frontal lobe; OL, occipital lobe; TL, temporal lobe.*

**FIGURE 4:**
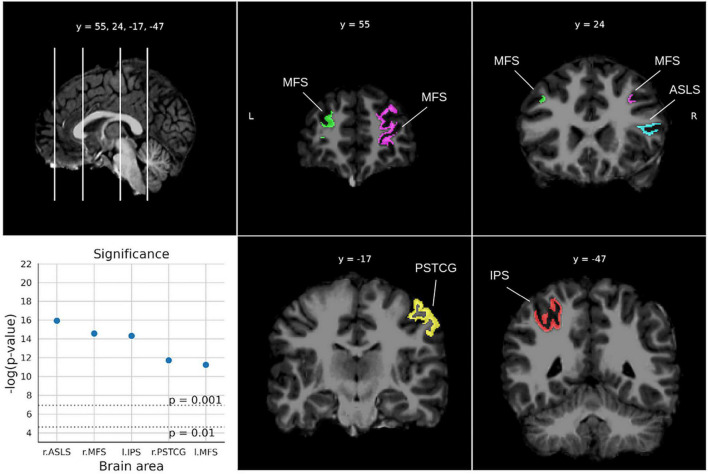
Significant areas for the Math fluency test with corresponding plots of the logarithmic *p*-values for each area. Coordinates are in MNI space. Brain areas: ASLS [Vertical ramus of the anterior segment of the lateral sulcus (or fissure)], MFS (Middle frontal sulcus), IPS (Intraparietal sulcus and transverse parietal sulci), PSTCG (Post-central gyrus).

**FIGURE 5:**
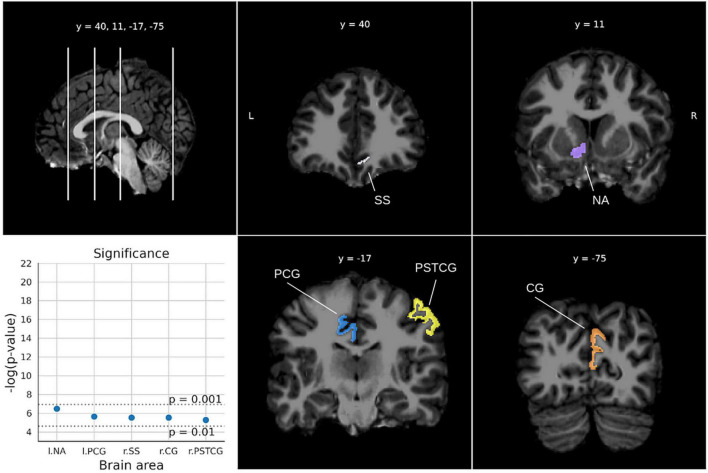
Significant areas for the calculation test with corresponding plots of the logarithmic *p*-values for each area. Coordinates are in MNI space. Brain areas: NA (Nucleus accumbens), PCG (Middle-posterior part of the cingulate gyrus and sulcus), SS [Suborbital sulcus (sulcus rostrales, supraorbital sulcus)], CG (Cuneus gyrus), PSTCG (Post-central gyrus).

For the Math fluency test (Figure 4), five areas were found below the two-sigma threshold with high significance (*ps* < 0.0014), involving the two brain hemispheres. In particular, the right lateral sulcus (Sylvian fissure), the left intraparietal sulcus and the right postcentral gyrus in the parietal lobe, and the middle frontal sulci bilaterally. For the Calculation test (Figure 5), five areas from both hemispheres were selected showing *ps*-values between 0.0052 and 0.0015. The most relevant region was the left accumbens area, followed by the left middle cingulate gyrus and sulcus, the right suborbital sulcus, the right cuneus gyrus, and the right postcentral gyrus.

**FIGURE 6:**
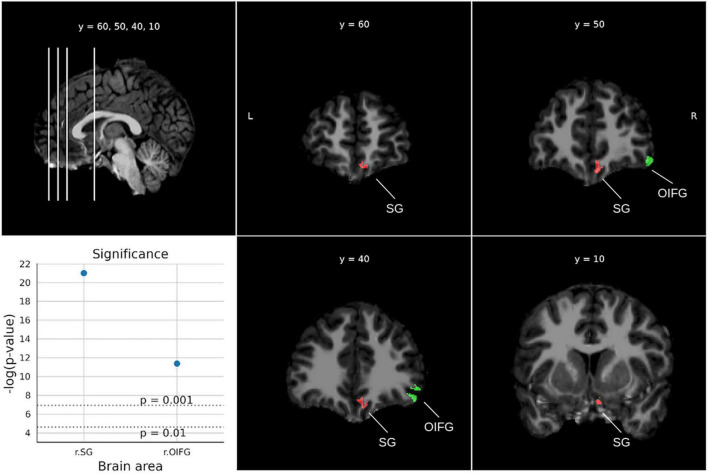
Significant areas for the applied problems test with corresponding plots of the logarithmic *p*-values for each area. Coordinates are in MNI space. Brain areas: SG (Straight gyrus, Gyrus rectus), OIFG (Orbital part of the inferior frontal gyrus).

For the Applied problems test (Figure 6), two significant areas were obtained (*ps* < 0.000013). In particular, the right rectus gyrus and the right inferior frontal gyrus.

**FIGURE 7:**
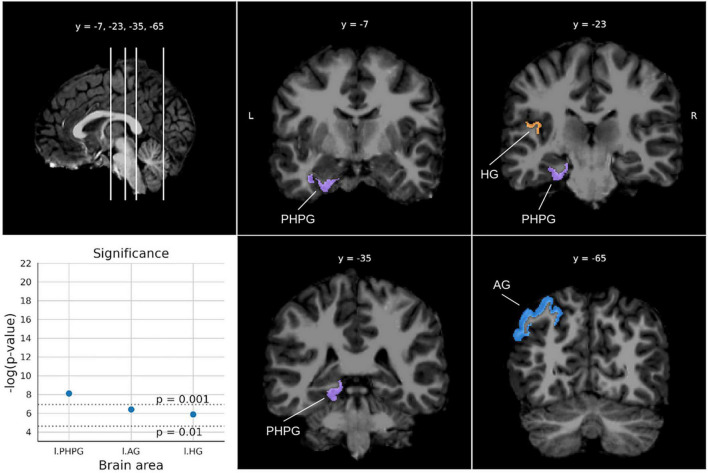
Significant areas for the Quantitative concepts test with corresponding plots of the logarithmic *p*-values for each area. Coordinates are in MNI space. Brain areas: PHPG (Parahippocampal gyrus, parahippocampal part of the middle occipito-temporal gyrus), AG (Angular gyrus), HG [Anterior transverse temporal gyrus (of Heschl)].

Finally, for the Quantitative concepts test (Figure 7), three areas from the left hemisphere were found with *p*-values below 0.0029. In particular, the parahippocampal gyrus was the most relevant area, followed by the angular gyrus and the Hesch gyrus.

**FIGURE 8:**
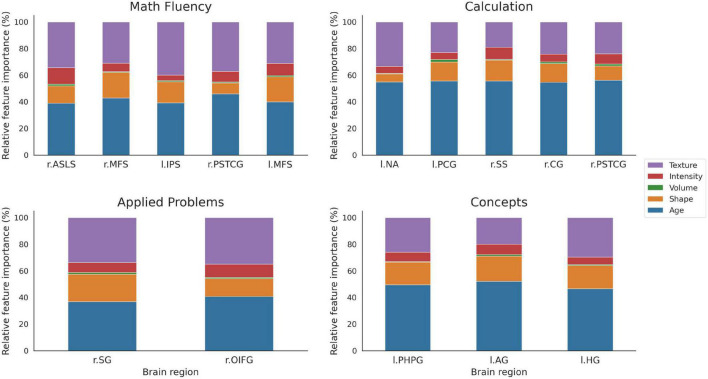
Relative percentage of variable importance presented by feature group for the selected brain areas. The total sum is rescaled to 100% to better appreciate differences between features. Note the smaller size of volume (green) compared with shape (orange) or texture (purple) features. Brain areas: ASLS [vertical ramus of the anterior segment of the lateral sulcus (or fissure)], MFS (middle frontal sulcus), IPS (intraparietal sulcus and transverse parietal sulci), PSTCG (post-central gyrus), NA (nucleus accumbens), PCG (middle-posterior part of the cingulate gyrus and sulcus), SS [suborbital sulcus (sulcus rostrales, supraorbital sulcus)], CG (cuneus gyrus), OIFG (orbital part of the inferior frontal gyrus), PHPG (parahippocampal gyrus, parahippocampal part of the middle occipito-temporal gyrus), AG (angular gyrus), HG [anterior transverse temporal gyrus [of Heschl)].

The relative *importance* variable is presented as given by the Gini *importance* index for each brain area selected in Table 3 (see Figure 8). The results were grouped by feature family to assess the relative *importance* of age, volume, shape, intensity, and texture, separately. The area volume, used mainly in VBM, was considered apart from shape features to assess its contribution independently. Among radiomics variables, texture features showed the greatest relative *importance* for all tests, which indicated their superior discriminative value. Importantly, volume represented less than 15% of the shape features contribution in all cases. Additionally, as a proxy for feature stability, radiomics with a great variability on Gini *importance* were highlighted. In detail, features with a variability above two standard deviations from the mean variability were flagged as outliers for every task. Four features were found as outliers across tasks. In order from most to least stable they were: 10th and 90th percentile, interquartile range (1st order), and long run emphasis (GLRLM).

## Discussion

The majority of sMRI studies that have explored the brain areas involved in mathematical abilities have primarily used volume-related measures. However, through the use of radiomics-based analyses we have shown here that texture features are the most important for the regression models explored, followed by shape features. Volume, however, is just one of the features belonging to the shape features family and provides a relatively small percentage of importance in the regression models in predicting children’s performance on math tests. This result suggests that other aspects of the brain areas, such as surface, length in a given direction, or the intensity pattern shown in the image, are even more important measures for predicting children’s mathematics performance. All these features together provided more information than volume alone. In fact, texture features have been found to be very important biomarkers for cognitive traits such as autism spectrum disorder (Chaddad et al., 2017) or schizophrenia (Park et al., 2020). The present study suggests that radiomics-based analyses can provide further detailed information about the medical image than more traditional measures. In fact, when compared with models that only took age into consideration, the current regression models that used radiomics features were able to reduce the MAE by a percentage between 5% (from 0.112 to 0.106 for Applied problems) and 18% (from 0.257 to 0.210 for Math fluency).

The results highlight the involvement of frontal areas, mainly in Math fluency and Applied problems, and parietal areas, mainly in Math fluency but also in Calculation and Concepts. Occipital areas were found in Calculation and temporal areas in Quantitative concepts. Finally, basal ganglia were associated with Calculation and areas of the limbic system to Calculation and Quantitative concepts. In line with previous neuroimaging studies, these areas seem to play a role in mathematical operations as well as in cognitive control and motivation (Arsalidou et al., 2018).

Math fluency is based on basic arithmetic operations that depend on recovery of number facts from long-term memory (Andersson, 2008), and therefore is expected to pose minimal demands on participants’ attentional/working memory capacity. Inferior frontal cortex, middle frontal cortex, and post-central gyrus (in the parietal cortex), mainly from the right hemisphere, are associated with mathematical abilities that are mainly based on automatized processes (Arsalidou et al., 2018). The left intraparietal sulcus plays also a central role in basic quantitative representation (Dehaene et al., 2004) and in addition and subtraction (Arsalidou and Taylor, 2011), representing the basis of the Math fluency test. In addition, the middle frontal cortex and the intraparietal sulcus may have shown a stronger relationship with this test given the attentional effort expected when children perform a test with important time constraints. Calculation is based on the rapid activation of the numerical magnitude of Arabic numerals, and arithmetic operations go in increasing order of complexity. As for Math fluency, important areas of the right frontal and parietal lobes usually associated with numerical automatized processes were also observed here (suborbital sulcus and post-central gyrus). More complex operations may be related to the posterior cingulate cortex, an area involved in memory retrieval (Rolls, 2019). In addition, the basal ganglia may be related to the motivational/affective components linked to the performance of this test. Accordingly, the nucleus accumbens has been associated with motivational behavior and effort regulation (Salamone, 1994; Nicola et al., 2005; Salamone et al., 2007). While the cuneus gyrus has been related to visual recognition of objects, the right cuneus gyrus has been specifically associated with the approximate calculation in children (Kucian et al., 2008). The results showed that the post-central gyrus was related to both Math fluency and Calculation. Previous studies have found that the cortical complexity of this region is associated with a high capacity for mathematical fluency (Polspoel et al., 2020). The activation of this somatotopic region responsible for the mouth, fingers and hands has been related to subvocalization and finger counting as a mathematical strategy (Kesler et al., 2006). Among the areas related to Calculation, only inferior frontal and occipito-temporal areas were also found in previous sMRI studies, showing reduced volume for children with low mathematical performance (De Smedt et al., 2019). The Applied problems test requires both to hold information in memory and to integrate new information with previous one (Lee Swanson, 2011; Pina et al., 2014), and therefore it is expected to impose more demands on executive control capacity than the two previous tests. Accordingly, the straight gyrus and orbital part of the inferior frontal gyrus seem to be associated with performance in this test. Less gray matter volume in the inferior frontal gyrus has been observed in children with poor mathematical abilities in sMRI studies (Peters and De Smedt, 2018). The straight gyrus is involved in attention control and it is functionally related with the orbital cortex (Nestor et al., 2015). Bilateral implication of the inferior frontal gyrus and the straight gyrus in arithmetic principles vs. computation has been reported (Liu et al., 2019). In addition, previous studies have shown the involvement of the frontal lobe, concretely bilateral activation of the inferior frontal gyri, in mathematical word problems (Prabhakaran et al., 2001). Finally, the Quantitative concepts test assesses mathematical knowledge (e.g., formulas and terms) and quantitative reasoning. Poor performance on this test is expected when participants show limited vocabulary or insufficient conceptual development (Pina et al., 2015). Accordingly, brain areas associated with performance in this test are expected to be lateralized in the left hemisphere, which could reflect a language mediation role (Grabner et al., 2007). Concretely, the parahippocampal part of the middle occipito-temporal gyrus is involved in remembering facts and rules (Squire et al., 2004) and it has been proposed that this area maintains memory representations during test performance (Rivera et al., 2005). The left angular gyrus located near the intraparietal sulcus has been related to the language required in some arithmetic operations that use verbal coding or are based on verbally stored knowledge (Grabner et al., 2007). Also, we observed the implication of Heschl gyrus, which corresponds to the primary auditory cortex. This area could be associated with this test because the items were read aloud, and children needed to be attentive to verbal information. Both parietal and occipito-temporal areas have been previously observed in sMRI studies in children with poor mathematical abilities (De Smedt et al., 2019).

Our results involved different brain areas depending on the processes required by the different mathematical abilities, and the main areas observed agree with those reported in both fMRI and sMRI studies. Importantly, the present study addressed the issue from a broader and novel perspective. First, a wide range of mathematical tests was considered, which differed not only in the specific mathematical abilities, but also in the degree of complexity of arithmetic operations and the demands on children’s attentional/working memory capacity. This makes the present study an important contribution to a better understanding of the brain areas that predict the diverse abilities required when people are confronted with mathematical facts. Second, areas related to specific mathematical abilities, mainly reported with fMRI, were found with sMRI by using radiomics.

Regarding children’s performance on mathematics tests, it should be taken into account that neurocognitive tests involve not only general ability but also capture a set of acquired abilities and skills (Colom et al., 2002). Consequently, children’s mathematical performance may depend on several factors in addition to the specific knowledge acquired during schooling (see Sánchez-Pérez et al., 2018b). Children may score higher on mathematics tests because they spend more time studying or because they have received extra tutoring, among other factors. Our results have shown the involvement of different brain areas as a function of the processes required by different mathematics tests, and the main areas observed here coincide with those reported in previous neuroimaging studies. Future studies should investigate the influence of these acquired skills on the correlations between brain structure and mathematical performance.

Two limitations have been identified for the present study. First, the sample size used is relatively small in comparison to other studies in precision medicine, although clearly superior to other neuroimaging approaches with children. Second, the 1.5T images used in this study have a relatively lower signal-to-noise ratio compared to those collected at 3T or higher, which could affect the robustness of texture features (Ammari et al., 2021). Despite the aforementioned limitations, to the best of our knowledge, this is the first study that analyzes mathematical performance in school-aged children with radiomics through sMRI images.

Briefly, the present study makes an important contribution to a better understanding of the brain areas that predict school-aged children’s performance in math tests. We extended the findings of previous sMRI related studies by using radiomics. Texture features rather than standard volumetric measures reached higher *importance* in predicting children’s performance on math tests. The open-sourced radiomics-based method proposed here can be easily automated and therefore potentially used by researchers and clinicians to perform a more exhaustive analysis of radiological studies that can help to better characterize brain anomalies associated with difficulties in learning mathematics.

## Data Availability Statement

The raw data supporting the conclusions of this article will be made available by the authors, without undue reservation.

## Ethics Statement

The studies involving human participants were reviewed and approved by the Comité de Ética, Universidad de Murcia. Written informed consent to participate in this study was provided by the participants’ legal guardian/next of kin.

## Author Contributions

VP, VC, and LJF conceptualized, designed the study, and wrote the manuscript. JG-S acquired the data. VP, VC, KL, SS, and LJF analyzed and interpreted the data. VC, SS, and KL prepared the figures. All authors read and approved the final version of the manuscript.

## Conflict of Interest

The authors declare that the research was conducted in the absence of any commercial or financial relationships that could be construed as a potential conflict of interest.

## Publisher’s Note

All claims expressed in this article are solely those of the authors and do not necessarily represent those of their affiliated organizations, or those of the publisher, the editors and the reviewers. Any product that may be evaluated in this article, or claim that may be made by its manufacturer, is not guaranteed or endorsed by the publisher.
