# Supplementary material for: Mathematical Abilities in School-Aged Children: A Structural Magnetic Resonance Imaging Analysis With Radiomics
Source: Front Neurosci. 2022 Apr 14;16:819069. doi: 10.3389/fnins.2022.819069 (PMC9047716; doi:10.3389/fnins.2022.819069)
Supplement: Supplementary file 1 [file Data_Sheet_1.docx]

Supplementary Material

**Table S1**. List of 100 Radiomics features considered during the analysis.

| Family | Index | Feature | Family | Index | Feature |
| --- | --- | --- | --- | --- | --- |
| Shape | 1 | Elongation | GLCM | 51 | InverseVariance |
|  | 2 | Flatness |  | 52 | MaximumProbability |
|  | 3 | LeastAxisLength |  | 53 | SumEntropy |
|  | 4 | MajorAxisLength |  | 54 | SumSquares |
|  | 5 | Maximum2DDiameterColumn | GLRLM | 55 | GrayLevelNonUniformity |
|  | 6 | Maximum2DDiameterRow |  | 56 | GrayLevelNonUniformityNormalized |
|  | 7 | Maximum2DDiameterSlice |  | 57 | GrayLevelVariance |
|  | 8 | Maximum3DDiameter |  | 58 | HighGrayLevelRunEmphasis |
|  | 9 | MeshVolume |  | 59 | LongRunEmphasis |
|  | 10 | MinorAxisLength |  | 60 | LongRunHighGrayLevelEmphasis |
|  | 11 | Sphericity |  | 61 | LongRunLowGrayLevelEmphasis |
|  | 12 | SurfaceArea |  | 62 | LowGrayLevelRunEmphasis |
|  | 13 | SurfaceVolumeRatio |  | 63 | RunEntropy |
|  | 14 | VoxelVolume |  | 64 | RunLengthNonUniformity |
| First order | 15 | 10Percentile |  | 65 | RunLengthNonUniformityNormalized |
|  | 16 | 90Percentile |  | 66 | RunPercentage |
|  | 17 | Energy |  | 67 | RunVariance |
|  | 18 | Entropy |  | 68 | ShortRunEmphasis |
|  | 19 | InterquartileRange |  | 69 | ShortRunHighGrayLevelEmphasis |
|  | 20 | Kurtosis |  | 70 | ShortRunLowGrayLevelEmphasis |
|  | 21 | Maximum | GLSZM | 71 | GrayLevelNonUniformity |
|  | 22 | MeanAbsoluteDeviation |  | 72 | GrayLevelNonUniformityNormalized |
|  | 23 | Mean |  | 73 | GrayLevelVariance |
|  | 24 | Median |  | 74 | HighGrayLevelZoneEmphasis |
|  | 25 | Minimum |  | 75 | LargeAreaEmphasis |
|  | 26 | Range |  | 76 | LargeAreaHighGrayLevelEmphasis |
|  | 27 | RobustMeanAbsoluteDeviation |  | 77 | LargeAreaLowGrayLevelEmphasis |
|  | 28 | RootMeanSquared |  | 78 | LowGrayLevelZoneEmphasis |
|  | 29 | Skewness |  | 79 | SizeZoneNonUniformity |
|  | 30 | TotalEnergy |  | 80 | SizeZoneNonUniformityNormalized |
|  | 31 | Uniformity |  | 81 | SmallAreaEmphasis |
|  | 32 | Variance |  | 82 | SmallAreaHighGrayLevelEmphasis |
| GLCM | 33 | Autocorrelation |  | 83 | SmallAreaLowGrayLevelEmphasis |
|  | 34 | JointAverage |  | 84 | ZoneEntropy |
|  | 35 | ClusterProminence |  | 85 | ZonePercentage |
|  | 36 | ClusterShade |  | 86 | ZoneVariance |
|  | 37 | ClusterTendency | GLDM | 87 | DependenceEntropy |
|  | 38 | Contrast |  | 88 | DependenceNonUniformity |
|  | 39 | Correlation |  | 89 | DependenceNonUniformityNormalized |
|  | 40 | DifferenceAverage |  | 90 | DependenceVariance |
|  | 41 | DifferenceEntropy |  | 91 | GrayLevelNonUniformity |
|  | 42 | DifferenceVariance |  | 92 | GrayLevelVariance |
|  | 43 | JointEnergy |  | 93 | HighGrayLevelEmphasis |
|  | 44 | JointEntropy |  | 94 | LargeDependenceEmphasis |
|  | 45 | Imc1 |  | 95 | LargeDependenceHighGrayLevelEmphasis |
|  | 46 | Imc2 |  | 96 | LargeDependenceLowGrayLevelEmphasis |
|  | 47 | Idm |  | 97 | LowGrayLevelEmphasis |
|  | 48 | Idmn |  | 98 | SmallDependenceEmphasis |
|  | 49 | Id |  | 99 | SmallDependenceHighGrayLevelEmphasis |
|  | 50 | Idn |  | 100 | SmallDependenceLowGrayLevelEmphasis |

The abbreviations stand for Gray Level Co-ocurrence Matrix (GLCM), Gray Level Run Length Matrix (GLRLM), Gray Level Size Zone Matrix (GLSZM) and Gray Level Dependence Matrix (GLDM). Details for interpreting these features as well as their definitions are provided in the PyRadiomics documentation: pyradiomics.readthedocs.io

**Feature selection details**

Figure S1 presents the features that survived the feature selection step for each significant area found in our analysis. A feature is categorized as selected (blue color) if it survived the feature selection for the majority of the 20 dataset partitions and as removed (orange color), otherwise. As explained in section 2.5, feature correlations were computed sequentially following the order in Table S1, which explains why more features were removed from the GLCM, GLRLM and GLDM feature families versus shape and first order features.


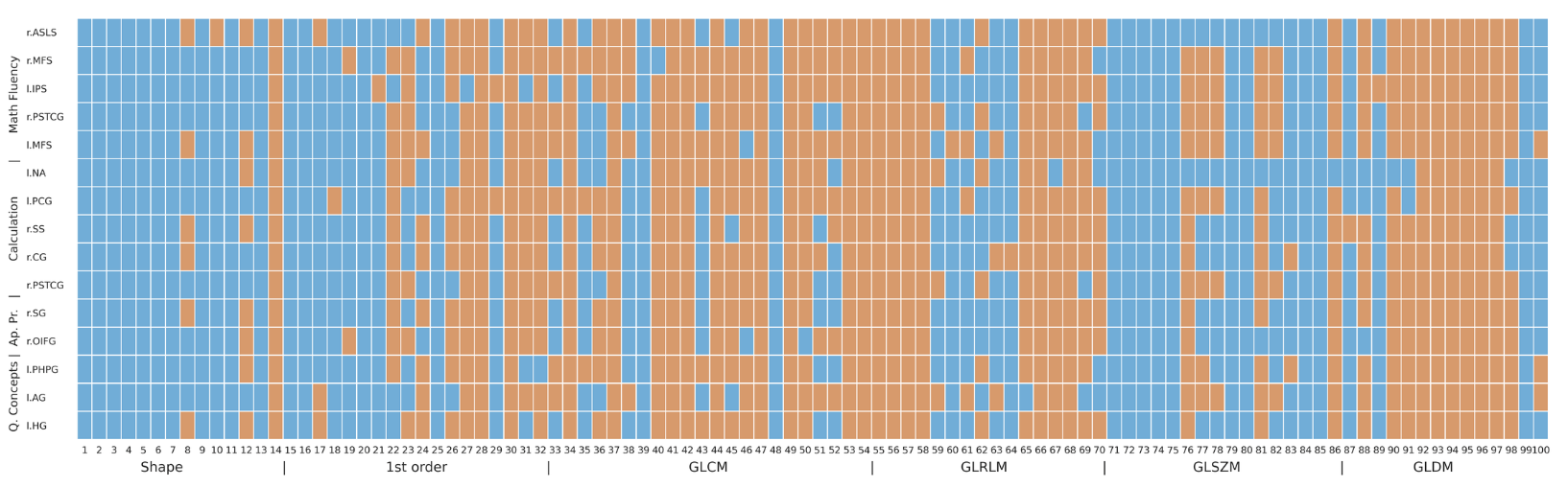


**Caption**

**Figure S1.** Visualization of the radiomic features that survived the feature selection step (in blue). Features that had a squared correlation coefficient higher than 0.8 were removed (in orange). Each row represents a significant area for the different tasks considered and each column corresponds to one feature as listed in Table S1.

**Table S2.** Mean absolute error (MAE) for predictive areas of mathematical performance in the four subtests of the WJ-III battery, with and without the feature selection step.

| Test | Atlas label | MAE  (with FS) | MAE  (without FS) |
| --- | --- | --- | --- |
| Math  fluency | 12140 | 0.210 | 0.224 |
|  | 12154 | 0.212 | 0.222 |
|  | 11157 | 0.212 | 0.213 |
|  | 12128 | 0.216 | 0.220 |
|  | 11154 | 0.217 | 0.224 |
| Calculation | 26 | 0.116 | 0.121 |
|  | 11108 | 0.118 | 0.121 |
|  | 12171 | 0.118 | 0.124 |
|  | 12111 | 0.118 | 0.124 |
|  | 12128 | 0.118 | 0.127 |
| Applied  problems | 12131 | 0.106 | 0.107 |
|  | 12113 | 0.112 | 0.112 |
| Quantitative  concepts | 11123 | 0.098 | 0.102 |
|  | 11125 | 0.100 | 0.102 |
|  | 11133 | 0.100 | 0.097 |
